# Non-coplanar VMAT plans for postoperative primary brain tumour to reduce dose to hippocampus, temporal lobe and cochlea: a planning study

**DOI:** 10.1259/bjro.20210009

**Published:** 2021-07-05

**Authors:** Eva Yi Wah Cheung, Kevin Ho Yuen Lee, Wilson Tin Long Lau, Amy Pik Yan Lau, Pak Ying Wat

**Affiliations:** 1 School of Medical Health Sciences, Tung Wah College, Hong Kong, China

## Abstract

**Objectives:**

This study aimed to compare radiotherapy plan quality of coplanar volumetric modulated arc therapy (CO-VMAT) and non-coplanar VMAT (NC-VMAT) for post-operative primary brain tumour.

**Methods:**

A total of 16 patients who were treated for primary brain tumours were retrospectively selected for this study. For each patient, identical CT sets with structures were used for both CO-VMAT and NC-VMAT planning. For CO-VMAT, one full arc and two coplanar half arcs were used. For NC-VMAT, one full coplanar and two non-coplanar half arcs with couch rotation of 315° or 45° were used. Dose constraints were adhered to the RTOG 0614, RTOG 0933 and TMH protocol. Dose volumetric parameters were collected for statistical analysis.

**Results:**

.

NC-VMAT achieved significant dose reduction in contralateral hippocampus, both temporal lobes and cochleae, and other OARs while the plan qualities remained the same. In particular, NC-VMAT decreased contralateral hippocampus mean dose by 1.67Gy. Similarly, the NC-VMAT decreased temporal lobe mean dose by 6.29Gy and 2.8Gy for ipsilateral and contralateral side respectively. Furthermore, it decreased cochlea mean dose by 5.34Gy and 0.97Gy for ipsilateral and contralateral side respectively. Overall, there was a reduction of 5.4% of normal brain tissue volume receiving low dose irradiation.

**Conclusion:**

The proposed NC-VMAT showed more favourable plan quality than the CO-VMAT for primary brain tumours, in particular to hippocampus, temporal lobes, cochleae and OARs located to the contralateral side of tumours.

**Advances in knowledge:**

For primary brain tumours radiotherapy, NC-VMAT can reduce doses to the hippocampus, both temporal lobes, and cochleae, as well as OARs located to the contralateral side of tumours.

## Introduction

Radiotherapy is currently chosen as a primary or post-operative adjuvant treatment modality for most primary brain tumour patients. It improves local tumour control and prolonged survival for patients when comparing to those who receive surgery alone.^1^ Using different radiotherapy technique is a new trend to treat cranial tumours, including IMRT and cyber-knife.^2,3^ Volumetric modulated arc therapy (VMAT) is a novel radiation technique, which deliver highly conformal radiation dose to the target volume using multiple intensity modulated arcs. Conformal dose can be achieved by combining variation of gantry rotation speed, treatment field size aperture and dose rate. It is a well-established treatment technique for all sites including head-and-neck regions,^4^ cranial regions,^5–7^ and other body sites.

Due to the trajectories of VMAT, irradiation to normal brain tissue is inevitable when treating primary brain tumours. Minimising dose to normal brain tissue is challenging in primary brain tumour VMAT planning, as it usually involves a large planned target volume. Non-coplanar radiotherapy uses a number of radiotherapy beams, that coincidence to the same isocentre, but not sharing the same geometric plane relative to the patients.^8^ Radiation dose reaches its maximum at the isocentre while entry dose can be distributed through different planes. Thus, dose to target volume remains the same while dose to OARs can be minimised.

Temporal lobe is one of the major lobes of cerebral cortex in a human brain. It comprises about 20% of the total volume of cerebrum. The superior temporal lobe demonstrates roles in language ability, whereas inferior and lateral portions of temporal lobe is important in confrontational naming. The medial part connects to medial structures including hippocampus which plays an important role in memory and perception.^9^ In addition, cochlear, which is responsible for hearing, is situated next to the temporal lobe. Previous studies have showed that patients may suffer from neurocognitive and auditory dysfunction after cranial irradiation.^7,10^ Hippocampal-sparing has been proposed in whole brain radiotherapy^5^ and for brain metastases^6^ and craniopharyngiomas^11^ using different radiotherapy techniques, including 7F IMRT, dual arc VMAT and coplanar VMAT. However, the application of co-planar and non-coplanar VMAT for multiple organ at risk sparing for primary brain tumours has not been well studied. The aim of this study has been set to compare the plan quality of coplanar VMAT (CO-VMAT) to that of non-coplanar VMAT (NC-VMAT) for primary brain tumours radiotherapy treatment in view of multiple organs-at-risk sparing.

## Methods and materials

### Patient selection

A total of 16 patients who were treated for primary brain tumours with IMRT technique were retrospectively selected from the clinical oncology department of Tuen Mun Hospital (TMH), New Territories West Cluster of Hospital Authority, Hong Kong. Eligibility criteria for this study was patients who had been diagnosed with primary brain tumour. Patients who had been diagnosed with brain metastases, treated with craniospinal irradiation, or having planning target volume (PTV) overlapping with both hippocampi were excluded. Ethics approval was obtained from the New Territories West Cluster Research Ethics Committee of the Hospital Authority, Hong Kong SAR (IRB/REC No: NTWC/REC/18113).

### Radiotherapy simulation

Simulation was performed with 3-mm-slice using Philips Brilliance 16 CT-scanner at TMH. Patients were simulated in supine position, with baseplate, headrest and thermoplastic cast, which was a standard radiotherapy setup for brain tumour treatment in TMH. Images were acquired without contrast agent. MRIs were acquired in private clinics and provided by the patients.

### Image co-registration and target and OAR delineation

Magnetic resonance (MR) images and CT images were co-registered before radiotherapy planning to improve the accuracy of delineation. PTV were contoured by the oncologists in TMH. Organs-at-risk (OARs), including brainstem, optic chiasm, cochleae, eyes, lens, optic nerves, spinal cord, temporomandibular joints (TM joint) and temporal lobes, were contoured by radiation therapists in TMH.

The hippocampi were contoured manually by investigators in this study, with assistance from radiation therapists in TMH, reference to the online RTOG contouring atlas (https://www.rtog.org/CoreLab/ContouringAtlases.aspx). Structures were approved by medical dosimetrists of TMH before radiotherapy planning.^12^ All MRI, CT images and structure sets were anonymised.

### Treatment planning: coplanar VMAT and non-coplanar VMAT

The planning software was Eclipse Radiotherapy Treatment Planning System, v. 15.6 (Varian Medical Systems, Palo Alto, CA). Dose prescription was 1.8 Gray (Gy) per fraction (1.8 Gy/fr), five fractions per week for 30 fractions to a total of 54 Gy. All plans were designed on Varian 21IX linear accelerator (Varian Medical System, Palo Alto, CA) equipped with the Varian High Definition 120 multileaf collimator (MLC), with 6 MV photon beams energy at a maximum dose rate of 600 monitor unit (MU) per minute. The dose rate of the treatment was set to 600 MU/min and the gantry speed was set at 4.8° per second in all plans. The type of MLC motion was sliding window. The single isocentre defined for all plans was set at the centre of the tumour. Field sizes were custom fitted by the arc geometry tool in Eclipse treatment planning system. The collimator rotation was set at 30° and 330° for clockwise and anti-clockwise arcs respectively to minimise inter-leaf transmission between MLC.

**Figure 1.**
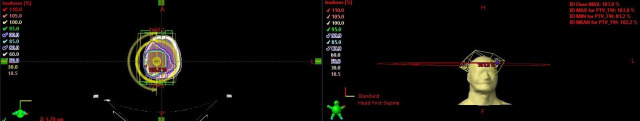
Arc configuration of CO-VMAT plan. CO-VMAT, coplanar volumetric modulated arc therapy

In the CO-VMAT plans, there were one full arc (179°−181°) and two half arcs. The couch angle for all arcs were kept at 0°. For cases with PTV located at the left side of the brain, the gantry angle for the two half arcs were set from 0° to 179° and 179° to 0°. While for cases with PTV on the right side of the brain, the gantry angle of the two half arcs were set from 0° to 181°and 181° to 0°. The arc configuration of CO-VMAT plan was shown in Figure 1.

**Figure 2.**
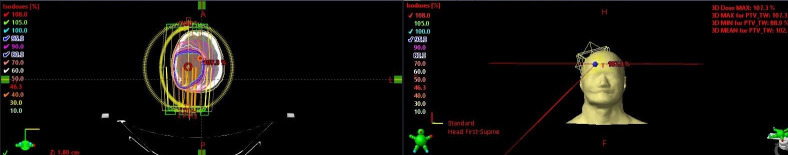
Arc and couch configuration of NC-VMAT plan. NC-VMAT, non-coplanar volumetric modulated arc therapy.

In the NC-VMAT plans, there were one full arc (179°−181°) and two half arcs. The couch angle for the full arc was at 0°. For cases with PTV located at the left side of the brain, the couch angle for the two half arcs was at 315°, and the gantry angle for the two half arcs were set from 0° to 179° and 179° to 0° . While for cases with PTV on the right side of the brain, the couch angle for the two half arcs was at 45°. The gantry angle for the two arcs were set from 0° to 181°and 181° to 0°. The arc and couch configuration of NC-VMAT plan was shown in Figure 2.

Both CO-VMAT and NC-VMAT plans were optimised using the progressive resolution optimiser (PRO3, v. 15.6, Varian Medical systems, Palo Alto, CA). The dose constraints applied during the optimisation process based on the RTOG 0614, 0933 and protocol from TMH. Details were listed in Tables 1 and 2. After optimisation, dose calculation were performed with Eclipse system using the Anisotropic Analytical Algorithm (AAA, v. 15.6, Varian Medical systems, Palo Alto, CA) with the grid size of 1.25 mm.

**Table 1.** Dose constraints for planning for CO-VMAT and NC-VMAT

Critical organ or organ-at risk	Dose constraint
Brainstem	Maximum dose <54 Gy
Optic chiasm	Maximum dose <54 Gy
Optic nerve	Maximum dose <54 Gy
Pituitary gland	Maximum dose <50 Gy
Eye balls	Maximum dose <50 Gy Mean dose <35 Gy
Lens	Maximum dose <8 Gy

CO-VMAT, coplanar volumetric modulated arc therapy; NC-VMAT, non-coplanar volumetric modulated arc therapy.

**Table 2.** Planning constraints

Structure	Dose constraint	Weight
PTV	Maximum dose <60 Gy Minimum dose <52 Gy	100 100
Eye balls	Maximum dose <7 Gy Mean dose <35 Gy	5
Lenses	Maximum dose <5 Gy	5
Hippocampus	Maximum dose <11 Gy D40% < 9 Gy	5 10
Hippocampus avoidance volume	N/A	N/A
Temporal lobe	N/A	N/A
Cochlear	N/A	N/A

PTV, planning target volume.

RTOG 0614, RTOG 0933 and TMH protocol

## Interplanner variability

All treatment plans were created by four planners with similar level of clinical radiotherapy treatment planning experience. Each planner was responsible for both CO-VMAT and NC-VMAT of four patients. To standardise the planning procedures and minimise the interplanner variabilities, three sets of CT were selected as the samples. The planners performed planning on these three sets of CT, the dose–volume metrics used in clinical practice were employed to access the planner’s performance.^13^ They all met the specified planning goals before starting to create treatment plans for this study.

### Evaluation of treatment plans

Plans were checked and approved by a qualified medical dosimetrist with the following criteria:

1. At least 98% of PTV should receive 100% of prescribed dose (54 Gy) and
2. Maximum dose of 2% of PTV should receive dose below 58.32Gy (108% of the prescribed dose).
3. Maximum point dose allowed within the plan (*i.e.* hotspots outside the PTV) should be below 108% of prescribed dose.
4. All dose constraints for OARs listed in Tables 1 and 2 should be met.

For each plan, the total monitor units were collected. For the PTVs, the maximum dose (D_max_), minimum dose (D_min_), mean dose (D_mean_), dose to 98%, 2%, 50% and 1cc of volume of PTV (D_98%_, D_2%_, D_50%_, and D_1cc_ ) were calculated. The normal brain volume irradiated by 30% of the prescribed dose (V_30%_) was also calculated.

The homogeneity index and conformation number, which were commonly used as a tool to evaluate radiotherapy plans, were measured.

Homogeneity index was calculated as:

HI = (D_2%_-D_98%_)/DP

where D_2%_ is the dose received by 2% (volume) of PTV, D_98%_ is the dose received by 98% volume of PTV and DP is the prescribed dose (54 Gy).^14^

Conformation number was calculated based on the following formula:

CI = (V_tpr_^2^)/ (VT ×V_pr_)

where V_tpr_ is the volume of PTV receiving prescribed dose (54 Gy), VT is the volume of PTV and V_pr_ is the volume enclosed by isodose of the prescribed dose (54 Gy).^15^

For pituitary, chiasm and brainstem, the D_max_ and D_mean_ were calculated. The dose–volumetric parameters (D_max_ and D_mean_) of both ipsilateral and contralateral temporal lobe, TM joint, optic nerve, lens, eye ball and cochlear were calculated. For hippocampus, D_max_, D_mean_ and D_40%_ were calculated.

### Statistical analysis

Statistical tests were performed using the IBM Statistical Package for the Social Sciences (SPSS) (v. 23.0). Non-parametric Wilcoxon signed-rank test was performed. In this study, *p*-values of less than 0.05 were regarded as statistically significant.^16^

All 32 plans were clinically checked and accepted by qualified medical dosimetrists. The results presented were based on these 32 approved plans.

## Results

### Patient demographics

16 patients were included in this study, with the age ranging from 27 to 70 years old. The male-to-female ratio was 9:7. 11 patients were diagnosed as glioblastoma multiform, and 5 were diagnosed as meningioma. All patients had received surgery before receiving radiotherapy. The size of the gross tumour ranged from 1.1 to 7.2 cm (in greatest dimension).

### Dose–volumetric of PTV and total monitor units

The dose–volumetric parameters of the PTV were shown in Table 3 for both CO-VMAT and NC-VMAT. The overall dose–volumetric parameters of CO-VMAT and NC-VMAT were similar, the difference were minimal and no significant difference were recorded. The mean MU of CO-VMAT and NC-VMAT were 474 ± 51.64 and 488 ± 73.05 respectively (*p* = 0.535).

**Table 3.** Dose–volumetric parameters of the PTV

DV parameters PTV	Co-planar VMAT (Mean ± S.D.)	Non-coplanar VMAT (Mean ± S.D.)	*p*-value
Maximum dose (Gy)	57.98 ± 0.36	58.16 ± 0.32	0.196
Mean dose (Gy)	55.25 ± 0.17	55.25. ± 0.41	1.00
Minimum dose (Gy)	43.59 ± 7.75	45.04 ± 6.67	0.23
D_98%_ (Gy)	54.17 ± 0.22	54.12 ± 0.30	0.569
D_2%_ (Gy)	56.30 ± 0.32	56.32 ± 0.33	0.244
D_50%_ (Gy)	55.27 ± 0.19	55.27 ± 0.13	0.605
D_1cc_ (Gy)	56.66 ± 0.29	56.72 ± 0.35	0.171
Homogeneity Index	0.0382 ± 0.0064	0.0392 ± 0.0069	0.535
Conformation number	0.939 ± 0.0181	0.941 ± 0.0127	0.301

PTV, planning target volume; VMAT, volumetric modulated arc therapy.

*p* value <0.05.

### Dose–volumetric parameters of centrally located OARs

The dose–volumetric parameters of centrally located OARs were shown in Table 4 for both CO-VMAT and NC-VMAT. The overall dose–volumetric parameters of pituitary, chiasm and brainstem of NC-VMAT were lower - than that of CO-VMAT, however, they were not significant different, except the D_mean_ of brainstem. The mean dose to brainstem of NCO-VMAT was 1.35 Gy less - than that of CO-VMAT (*p* = 0.02).

**Table 4.** Dose–volumetric parameters of centrally located OARs

DV parameters Centrally located OARs		Co-planar VMAT (Mean ± S.D.)	Non-coplanar VMAT (Mean ± S.D.)	*p*-value
Pituitary	D_max_ (Gy)	9.73 ± 0.36	6.776 ± 4.80	0.152
	D_mean_ (Gy)	15.34. ± 5.34	7.99 ± 8.13	0.087
Chiasm	D_max_ (Gy)	20.53 ± 11.96	18.36 ± 11.76	0.255
	D_mean_ (Gy)	11.64 ± 7.33	8.93 ± 5.05	0.07
Brainstem	D_max_ (Gy)	23.3 ± 13.93	22.24 ± 11.87	0.877
	D_mean_ (Gy)	6.04 ± 3.94	4.69 ± 2.56	0.02^a^

OAR, organ at risk; VMAT, volumetric modulated arc therapy.

a *p*-value <0.05.

### Dose–volumetric parameters of other OARs – ipsilateral side

The dose–volumetric parameters of other OARs located on ipsilateral sideof the tumour were shown in Table 5 for both CO-VMAT and NC-VMAT. The dose–volumetric parameters of hippocampus, optic nerve, lens, eye ball of CO-VMAT were higher than that of NC-VMAT, however, they were not significant different. The D_mean_ of temporal lobe, cochlear and TM joint were higher in CO-VMAT than that of NC-VMAT, by 5.57 Gy (*p* = 0.006), 5.34 Gy (*p* = 0.002) and 10.77 Gy (*p* = 0.0001) respectively.

**Table 5.** Dose–volumetric parameters of other OARs – ipsilateral side

DV parameters Ipsilateral OARs		Co-planar VMAT (Mean ± SD)	Non-coplanar VMAT (Mean ± SD)	*p-*value
Hippocampus	D_max_ (Gy)	42.07 ± 16.94	39.71 ± 20.49	0.679
	D_mean_ (Gy)	26.90 ± 16.80	24.96 ± 18.56	0.483
	D_40%_	25.07 ± 16.77	16.56 ± 20.29	0.796
Temporal lobe	D_max_ (Gy)	54.76 ± 7.14	52.77 ± 13.48	0.307
	D_mean_ (Gy)	31.80 ± 12.78	25.51 ± 17.54	0.006^*a*^
Cochlear	D_max_ (Gy)	16.05 ± 14.44	9.41 ± 14.09	0.002^*a*^
	D_mean_ (Gy)	11.96 ± 11.68	6.62 ± 9.74	0.002^*a*^
TM joint	D_max_ (Gy)	20.30 ± 9.74	5.47 ± 8.14	0.0001^*a*^
	D_mean_ (Gy)	14.12 ± 8.64	3.35 ± 4.12	0.0001^*a*^
Optic nerve	D_max_ (Gy)	14.50 ± 8.48	13.49 ± 11.29	0.717
	D_mean_ (Gy)	10.33 ± 5.47	9.34 ± 7.08	0.756
Lens	D_max_ (Gy)	5.70 ± 1.11	5.61 ± 1.78	0.679
	D_mean_ (Gy)	4.38 ± 1.06	4.29 ± 1.62	0.277
Eye ball	D_max_ (Gy)	16.18 ± 5.63	16.10 ± 5.09	0.959
	D_mean_ (Gy)	7.12 ± 1.91	6.60 ± 2.97	0.438

OAR, organ at risk; TM, temporomandibular; VMAT, volumetric modulated arc therapy.

a *p*-value <0.05.

### Dose–volumetric parameters of other OARs – Contralateral side

The dose–volumetric parameters of other OARs located on contralateral side of the tumour were shown in Table 6 for both CO-VMAT and NC-VMAT. The dose–volumetric parameters of hippocampus, temporal lobe, TM joint, optic nerve, lens, eye ball and cochlear of CO-VMAT were significantly higher than that of NC-VMAT (all with *p* < 0.05). For the hippocampus, every dose–volumetric parameter of CO-VMAT was higher than those of NC-VMAT. The D_max_, D_mean_ and D_40%_ of CO-VMAT were 17.39 Gy, 6.16 Gy and 5.75 Gy, while those of NC-VMAT were 13.46 Gy (*p* = 0.05), 4.49 Gy (*p* = 0.001) and 3.8 Gy (*p* = 0.002) respectively. For temporal lobe, the D_max_ and D_mean_ of CO-VMAT were 20.12 Gy and 6.48 Gy, while those of NC-VMAT were 11.56 Gy (*p* = 0.0001) and 3.68 Gy (*p* = 0.0001). For cochlear, the D_max_ and D_mean_ of CO-VMAT were 2.88 Gy and 2.30 Gy, while those of NC-VMAT were 1.61 Gy (*p* = 0.0001) and 1.33 Gy (*p* = 0.0001).

**Table 6.** Dose–volumetric parameters of other OARs – contralateral side

DV parameters Contralateral OARs		Co-planar VMAT (Mean ± SD)	Non-coplanar VMAT (Mean ± SD)	*p*-value
Hippocampus	D_max_ (Gy)	17.39 ± 8.57	13.46 ± 10.28	0.05^*a*^
	D_mean_ (Gy)	6.16 ± 2.44	4.49 ± 2.00	0.001^*a*^
	D_40%_	5.75 ± 2.64	3.80 ± 1.73	0.002^*a*^
Temporal lobe	D_max_ (Gy)	20.12 ± 6.34	11.56 ± 4.93	0.0001^*a*^
	D_mean_ (Gy)	6.48 ± 2.76	3.68 ± 1.76	0.0001^*a*^
Cochlear	D_max_ (Gy)	2.88 ± 2.13	1.61 ± 0.80	0.0001^*a*^
	D_mean_ (Gy)	2.30 ± 1.57	1.33 ± 0.57	0.0001^*a*^
TM joint	D_max_ (Gy)	2.10 ± 1.72	1.17 ± 0.63	0.0001^*a*^
	D_mean_ (Gy)	1.58 ± 1.15	0.87 ± 0.40	0.0001^*a*^
Optic nerve	D_max_ (Gy)	7.61 ± 5.8	4.54 ± 2.55	0.001^*a*^
	D_mean_ (Gy)	5.88 ± 4.16	3.53 ± 2.10	0.0001^*a*^
Lens	D_max_ (Gy)	4.61 ± 2.07	2.94 ± 1.40	0.001^*a*^
	D_mean_ (Gy)	3.61 ± 1.81	2.29 ± 1.12	0.001^*a*^
Eye ball	D_max_ (Gy)	10.60 ± 4.27	6.08 ± 2.71	0.001^*a*^
	D_mean_ (Gy)	4.64 ± 2.37	2.84 ± 1.38	0.0001^*a*^

OAR, organ at risk; TM, temporomandibular; VMAT, volumetric modulated arc therapy.

a *p*-value <0.05.

### Normal brain tissue low dose volume (V_30%_)

The normal brain volume received low dose (30% of prescribed dose, *i.e.* 16.2 Gy) in CO-VMAT and NC-VMAT were 1230.93 cm^3^ and 1166.18 cm^3^ respectively (*p* = 0.044).

## Discussion

### Significance of the study

In this study, NC-VMAT showed similar dose–volumetric parameters in PTV and plan quality, but more favourable dose–volumetric parameters in OARs when comparing to CO-VMAT. The dose–volumetric parameters of every OARs located in the contralateral side of tumour of the NC-VMAT were less than those of CO-VMAT, indicated that the delivered doses to OARs of contralateral side were always less than those of CO-VMAT. While for OARs located in the ipsilateral side of tumour, the temporal lobe, TM joint and the cochlear were irradiated with significantly less dose in NC-VMAT when comparing to CO-VMAT.

### Hippocampus sparing

Hippocampus sparing radiotherapy has been proposed, with the clinical trials results of Phase III RTOG 0614 and Phase II RTOG 0933, to improve the delayed recall in patients who received whole brain radiotherapy. The prescribed dose to whole brain radiotherapy is 30 Gy. However, the prescribed dose to primary brain tumours is 54 to 60 Gy. It is more challenging to reduce the radiation dose to nearby OARs in radiotherapy to primary brain tumours. Gondi et al (2014) suggested that dose to 40% (D_40%_) of bilateral hippocampi greater than 7.3 Gy may lead to long-term impairment in delayed recall after irradiation.^17^ In this study, 9 out of 16 patients having the whole ipsilateral hippocampus overlapping with the PTV. Thus, dose to ipsilateral hippocampus was highly inevitable. The D_40%_ of ipsilateral hippocampus were 25.07 Gy and 16.56 Gy in CO-VMAT and NC-VMAT respectively. While for D_40%_ of contralateral hippocampus, both CO-VMAT and NC-VMAT achieved lower than 7.3 Gy (5.75 Gy and 3.8 Gy respectively), with NC-VMAT showed significantly lower dose than that of CO-VMAT. According to the Tsai et al (2015) study, the verbal memory damage was associated with the radiation dose and irradiation volume of hippocampus.^10^ NC-VMAT delivered lower dose to both hippocampi and it might help their functional preservation. The application would be beneficial to children and young adults with brain tumours, as they would have longer life expectancy after therapy.^18^

### Temporal lobe sparing

Temporal lobe injury had long been discussed as a radiation induced side-effect of nasopharyngeal carcinoma. Kazda et al. (2014) suggested that temporal lobe received a mean dose of 45 Gy or higher was associated with the decline of longitudinal IQ.^19^ In addition, Hsiao et al (2010) study concluded that dose to the mean temporal lobe (D_mean_) should be lower than 36 Gy to minimise the neurocognitive damages.^20^ In our results, D_mean_ to ipsilateral temporal lobe delivered by CO-VMAT and NC-VMAT were 31.8 Gy and 25.5 Gy respectively, where NC-VMAT was significantly lower than that of CO-VMAT by 19.8%.

### Cochlear sparing

Radiation-induced auditory toxicity includes sensorineural hearing loss. A mean dose of 47 Gy to the cochlea would result in developing of severe high frequency sensorineural hearing loss.^21^ In this study, all dose–volumetric parameters of both ipsilateral and contralateral cochlea were below 47 Gy. The NC-VMAT reduced dose to ipsilateral and contralateral cochlear by 41.3% and 44% when compared to CO-VMAT.

### Low dose irradiation to normal brain tissue

Irradiation to normal brain tissue may lead to decline in microglial activation^22^ and resulted to cognitive impairment even though there is no noticeable damage to specific brain tissue.^23^ Our results showed that the volume of normal brain tissue receiving low dose in NC-VMAT was 5.4% smaller than that in CO-VMAT. The finding was coherent with the result from Audet et al (2011) study, which proved that using non-coplanar arcs could reduce low dose received by normal brain tissue.^24^

### Delivery efficiency

It has been reported that the delivery time of non-coplanar plan is longer when comparing to coplanar plan for high-grade glioma radiotherapy treatment.^25^ The time required to deliver a 1.8 Gy fraction of cranial treatment is around 2 min using coplanar VMAT.^26^ For the NC-VMAT in this study, which required one couch rotation, needed additional 1 min if moved manually, or 20 sec if moved automatically using dynamic couch rotation. The delivery time remains short using NC-VMAT in this case.

### Patient safety

One of the main concerns for NC-VMAT is the risk of collision between the linear accelerator and the patient immobilisation devices. The potential collision depends on the tumour location, immobilisation devices, patient size and degree of couch rotation.^27^ In this study, 45 degree of couch rotation increased the risk of collision. However, the risk of collision can be minimised with a couch-top extension installed onto the superior part of the couch. All the NC-VMAT plans in current study have been simulated in the linear accelerator and no collision has been observed. To implement non-coplanar treatment in clinical setting, the immobilisation devices and predefined trajectories have to be checked and approved prior to - plan optimisation. Therefore, plan setup trial and manual couch rotation are recommended for every new non-coplanar treatment. With the support of collision prediction techniques and patient modelling, the risk of collision can be minimised.

### Limitation of the study

One of the limitations of this study was the nature of brain tumours, which could be originated in various location of the brain with a variety of shapes and sizes. The location of PTV and its distance from hippocampus varied between patients. Dose to hippocampi could be minimised if the PTV locates far from the hippocampi. In Korkmaz Kirakli and Oztekin (2017) study, the distance between multiple brain metastases and hippocampi were measured to evaluate the risk of recurrence after whole brain radiotherapy^28^. While in this study, primary single tumour was treated. The plan optimisation was straightforward with prescribed dose to PTV and as low as possible dose to OARs. The normal braintissue (non-PTV) received much lower dose than whole brain radiotherapy. The distance between the PTV and the contralateral hippocampus was not considered in this study. In hippocampus sparing radiotherapy, the region surrounding spared hippocampus might receive lower dose compared with other parts of the brain or the PTV. Although it had been reported that hippocampus sparing radiotherapy should be a safe technique to apply, there was an increase in absolute risk of recurrence of 2% in a hippocampus sparing irradiation^29^. For future research, distance between PTV and the hippocampus may be included as a parameter to evaluate the risk of recurrence of the disease.

The settings of the non-coplanar trajectories in this study may also be an issue. Compared with the study conducted by Uto et al (2016)^11^ , they obtained significant improvement in homogeneity index in NC-VMAT plans compared to CO-VMAT plans^14^ while we had no significant difference in homogeneity index. This may be due to the plan settings which we used collimator rotation of 30° or 330° in clockwise and anti-clockwise arcs respectively, but they used 0° collimator rotation in all arcs.

The sample size of this study was small. We tried to minimise the small sample size effect by producing the CO-VMAT plan and NC-VMAT plan based on all the patients we collected.

In this study, all the plans were designed on a Varian 21IX linear accelerator, which did not equip with jaw tracking capability. The machine was chosen based on the availability in the real clinical setting in TMH. Snyder et al (2014) study proved that jaw tracking helped in reducing dose to spinal cord in both IMRT and VMAT in spine radiosurgery without a change in calculation accuracy.^30^ Further reduction in the radiation dose to OARs and normal brain tissue (non-PTV brain volume) might be achieved in both CO-VMAT and NC-VMAT with jaw tracking capability, while the investigation could be done in the future study.

In current study, the couch angle of 315° or 45° was chosen in NC-VMAT for tumour located at the left side and right side of the brain respectively. While we focused on demonstrating the OARs sparing effects in NC-VMAT, it would be worthwhile to include several beam configurations of NC-VMAT for comparison in future studies, so as to choose the best configurations among them to achieve the best OARs sparing effects for brain tumour patients.

## Conclusion

For primary brain tumours radiotherapy, the proposed NC-VMAT technique achieved treatment qualities comparable to the CO-VMAT plans. The NC-VMAT reduced dose to the brainstem, both temporal lobes, TM joints and cochleae. Also, it reduced dose to contralateral hippocampus, optic nerve, lens and eyeball. Thus, the NC-VMAT can be a direction of new standard of care for brain tumour patients,to preserve their neurocognitive and OARs functions after radiotherapy.
